# The Effect of *Broussonetia papyrifera* Silage on the Growth Performance, Blood Physiological Parameters, Serum Biochemical Parameters, Immune Response, Antioxidant Capacity, and Rumen Bacteria of Kazakh Lamb

**DOI:** 10.3390/ani15010078

**Published:** 2025-01-01

**Authors:** Xiaokai Zheng, Yixiang Wang, Shuangming Li, Yingchao Sun, Guoqing Hou, Rongzheng Huang, Fanfan Zhang

**Affiliations:** 1 College of Animal Science and Technology, Shihezi University, Shihezi 832003, China; zhengxiaokaiyue@163.com (X.Z.); wyx18369499053@163.com (Y.W.); lishuangming0410@163.com (S.L.); 17633866730@163.com (Y.S.); 2 Institute of Agriculture Science, Seventh Division of Xinjiang Production and Construction Corps, Kuitun 833200, China; 18230191323@163.com

**Keywords:** *Broussonetia papyrifera*, growth performance, serum immunity, serum antioxidant, rumen bacteria

## Abstract

Numerous studies have demonstrated that *Broussonetia papyrifera* is an unconventional feed resource with significant developmental potential. This research aimed to explore the effects of *Broussonetia papyrifera* silage on the growth performance, blood parameters, immunity, antioxidation, cytokine levels, and rumen bacterial composition of Kazakh lamb. Forty healthy male Kazakh lambs, aged 5 months and weighing 30.12 ± 1.14 kg, were randomly divided into control and experimental groups, each consisting of four replicates (five lambs per replicate). The control group was fed a basal diet, while the experimental group received a diet supplemented with 20% *Broussonetia papyrifera* silage (dry matter basis). Following a 10-day pre-feeding period, a 60-day formal experiment was conducted. The results indicated no significant difference in growth performance between the experimental and control groups. However, compared to the control group, the use of *Broussonetia papyrifera* silage significantly reduced (*p* < 0.05) neutrophil, lymphocyte, and eosinophil counts, as well as creatinine levels in the blood. Furthermore, *Broussonetia papyrifera* silage (*p* < 0.01) enhanced total serum antioxidant capacity, superoxide dismutase, catalase, glutathione peroxidase, immunoglobulin A, immunoglobulin M, immunoglobulin G, interleukin-2, interleukin-6, and interleukin-8, and decreased malondialdehyde and interleukin-4 levels. Additionally, the use of *Broussonetia papyrifera* silage increased the diversity and richness of the rumen bacterial community, notably enhancing the relative abundance of *Firmicutes* such as *Rikenellaceae_RC9_gut_group* and *Christensenellaceae_R-7_group*. In conclusion, feeding Kazakh lamb with *Broussonetia papyrifera* silage (20% DM) did not adversely affect their growth performance but improved their immunity and antioxidant capacity and enhanced the relative abundance of beneficial bacteria in the rumen, thereby promoting animal health.

## Simple Summary

In recent years, there has been a severe shortage of crude protein feed resources for ruminants due to the rapid growth of the animal industry, with traditional feed resources such as alfalfa and soybean meal unable to meet this challenge because of production and price factors. Therefore, it is necessary to explore alternative protein feed resources. *Broussonetia papyrifera* silage, as a non-conventional protein feed resource, has significant potential. Thus, this study evaluated the effectiveness of *Broussonetia papyrifera* silage feed for ruminants by investigating its effect on the growth performance, blood parameters, immune response, antioxidant function, and rumen microbial community composition of Kazakh lamb. The findings indicate that *Broussonetia papyrifera* silage does not adversely affect the growth performance of these lambs; instead, it shows promise in enhancing immune response and antioxidant capacity while promoting a balanced rumen microbial ecology.

## 1. Introduction

With the continuous expansion of animal husbandry, the demand for feed resources is steadily increasing. Among these resources, the scarcity of protein feed represents a primary constraint on high-quality animal production. The development of non-traditional feed sources can provide additional protein resources for animal husbandry [1].

*Broussonetia papyrifera* is a large deciduous tree belonging to the Moraceae family that is widely distributed in East and South Asia, including countries such as China and India [2]. Its branches and foliage are rich in various anti-inflammatory and antioxidant compounds, including phenols and alkaloids. Moreover, its high protein content, rapid growth rate, strong stress resistance, and abundant biomass make it an invaluable resource for livestock production [3]. Based on these attributes, the Institute of Botany at the Chinese Academy of Sciences has successfully developed a new strain of *B. papyrifera* that retains fundamental traits while boasting a well-balanced amino acid profile and impressive protein content, thereby earning recognition as a highly promising source of protein feed [3].

Currently, silage is the primary processing method for *Broussonetia papyrifera*. Although some nutrients may be diminished during fermentation, this process effectively reduces anti-nutritional factors such as tannins, polyphenols, and silicates [1,3]. Furthermore, the palatability of silage is enhanced, thereby promoting livestock digestion. Previous studies have shown that incorporating 24% dry matter of *B. papyrifera* silage into diets for young Hu sheep fosters both physical growth and immune function [4]. Moreover, the inclusion of 10% dry matter of *B. papyrifera* silage has been found to significantly enhance growth performance and meat quality in adult Hu sheep [5]. Replacing alfalfa hay (600 g/kg) with 33% (198 g/kg) of *B. papyrifera* silage could reduce feeding costs and enhance the antioxidant capacity of Saanen dairy goats, although it had no effect on their growth and organ development [6].

Additionally, dietary supplementation of 40% dry matter of *B. papyrifera* silage increased the relative abundance of *Christensenellaceae_R-7_group* in the rumens of Hu sheep, thereby influencing the unsaturated fatty acid composition in their longissimus dorsi muscles [7]. As an unconventional protein source, *B. papyrifera* silage is extensively utilized in ruminant husbandry, offering promising applications for improving productivity, enhancing animal well-being, and reducing breeding costs [8,9,10]. The impact of *B. papyrifera* silage varies among different lamb breeds. However, the effect of *B. papyrifera* silage on the growth performance of Kazakh lamb remains uncertain.

Kazakh lambs are a Chinese breed distinguished by their meat and fat production, coarse wool, rapid growth, adaptability to rough feed, and resilience in extreme cold conditions. The aim of this study was to evaluate the efficacy of *B. papyrifera* silage (20% DM) as an alternative feed resource for Kazakh lamb by analyzing growth performance, blood parameters, immune function, antioxidant capacity, and bacterial diversity in their rumen.

## 2. Materials and Methods

The animal research protocol was approved by the Biological Ethics Committee of Shihezi University (Shihezi, China) in March 2023, with the approval number A2023-129.

### 2.1. Preparation of Silage from Broussonetia papyrifera

The experimental site was located at the *Broussonetia papyrifera* demonstration base of the Seventh Agricultural Science Institute in Xinjiang Province (44°20′ N 83°51′ E, elevation 450 m). The *B. papyrifera* was harvested on 20 April 2023, at a height of 120 cm, leaving a stubble height of approximately 10 cm; the entire plant was then cut down and chopped to a length of 2–3 cm. The plant was inoculated with 1 × 10^5^ CFU/g of *Lactiplantibacillus plantarum* and mixed thoroughly [3]. Subsequently, the *B. papyrifera* silage was wrapped and sealed, with each package weighing approximately 80 kg. The silage underwent fermentation for 60 days at a storage temperature of 24 °C. The nutrient indicators were analyzed after ensiling and are presented in Table 1 as percentages of dry matter.

### 2.2. Animal Experimentation and Experimental Design

A total of 40 male Kazakh lambs (castrated rams), approximately 5 months old and weighing 30.12 ± 1.14 kg each, were selected for this study. These lambs were randomly divided into two groups, each consisting of 4 replicates (5 lambs per replicate). The control group was provided with a basal diet, while the experimental group received a total mixed ration consisting of *B. papyrifera* silage (20% dry matter). The formulation of the experimental diet adhered to the Chinese Sheep Feeding Standard (NY/T 816-2021) to meet the nutritional requirements during their growth phase. Detailed information regarding the composition and nutrient levels of the experimental diet is presented in Table 2, indicating a rough ratio of crude to refined ingredients of 4:6. The feeding period lasted for 10 days, followed by an additional experimental duration of 60 days. This experiment was conducted in Jinghe County, Xinjiang Province (82°30′49.20″ N; 44°31′19.58″ E; elevation 384 m). Each test lamb was individually housed in a pen according to its respective replicate and had ad libitum access to water throughout the study period. The sheep were fed twice daily at designated times (08:00 and 18:00), with feed leftovers limited to approximately 5%. The nutrient levels of corn silage in feed are shown in Table 1.

### 2.3. Sample Collection

On days 30 and 60 of the feeding trial, 10 lambs from each group were selected based on their similarity in weight to the group’s average. Following a 12 h fast, blood samples were collected from their jugular veins using heparin-coated vacuum blood collection tubes and anticoagulant vacuum blood collection tubes, both procured from Hebei Kangweishi Medical Technology Co., Ltd., Shijiazhuang, China. Two blood samples were obtained from each lamb (one per tube), with one sample designated for physiological blood tests. The anticoagulant vacuum blood collection tubes were inclined at a 45-degree angle for 30 min for gravity separation, followed by centrifugation at 3500 rpm for 10 min to separate the serum. The upper layer of serum was then stored at −80 °C for future use in serum tests [8]. On the 30th and 60th days of the experiment, 5 out of the 10 trial lambs were selected for rumen fluid collection using a rumen cannula. Before collection, the cannula was cleaned and disinfected with sterile saline. The lambs were then restrained, and rumen contents were gently aspirated using a sterile syringe through the cannula. The collected rumen fluid was filtered through 4 layers of gauze, divided into two 50 mL centrifuge tubes, immediately placed in liquid nitrogen, and then stored at −80 °C for rumen microbial determination.

### 2.4. Measurement of Indicators

#### 2.4.1. Feed Nutrient Level

Dry matter was determined according to AOAC standard procedures. Calcium (Ca) content was assessed following the AOAC official method (AOAC 968.08; Official Method for Determination of Calcium Content. AOAC International: Rockville, MD, USA, 2000). Phosphorus (P) content was determined using the AOAC official method (AOAC 965.17; Official Method for Determination of Phosphorus Content. AOAC International: Rockville, MD, USA, 2000). Acid detergent fiber (ADF) content was measured according to the AOAC official method (AOAC 973.18; Official Method for Determination of Acid Detergent Fiber Content. AOAC International: Rockville, MD, USA, 2000). The nitrogen content of the feed was determined using the Kjeldahl method, and neutral detergent fiber (NDF) was assessed according to the method described by Van Soest [11].

#### 2.4.2. Growth

On the 1st day and 60th day of the experiment, the weight of the test lamb was measured in the morning on an empty stomach. During the experiment, the daily feed intake and leftover feed were recorded, and the average daily weight gain (ADG), average daily feed intake (ADMI), and feed-to-gain ratio (F/G) were calculated after the experiment.

During the experiment, the initial weight and final weight of each lamb were recorded, and the total dietary consumption, ADG, ADFI, and F/G of each lamb were measured during the experiment. At the end of the experiment, 6 test lambs were randomly selected from each group and slaughtered, and the electrical stunning method was used for slaughter. The slaughter rate was calculated. The calculation formula is as follows:

ADG/(kg/d) = Final weight − Initial weight/total test days,

ADFI/g = total dietary consumption during the experiment/total test days,

Feed-to-meat ratio (F/G) = ADFI/ADG.

#### 2.4.3. Blood Physiology and Determination of Serum Biochemical Indicators

The detection of blood physiological indices primarily included the following: white blood cell count (WBC), neutrophil count (NEU), lymphocyte count (LYM), eosinophil count (EOS), red blood cell count (RBC), hemoglobin concentration (HGB), hematocrit (HCT), mean corpuscular volume (MCV), mean corpuscular hemoglobin content (MCH), mean corpuscular hemoglobin concentration (MCHC), platelet count (PLT), mean platelet volume (MPV), platelet distribution width (PDW), and plateletcrit (PCT). The XN-3000 blood cell analyzer and its accompanying reagents from Sysmex, Japan, were used to determine these physiological blood test indicators from whole-blood samples. Biochemical indicators in blood serum include albumin (ALB), total protein (TP), globulin (GLOB), total bilirubin (TB), aspartate aminotransferase (AST), alanine aminotransferase (ALT), amylase (AMY), creatine kinase (CK), creatinine (Crea), urea (UREA), glucose (Glu), and triglycerides (TG). After thawing the serum sample, it was transferred to a Smart Animal Biochemistry Analyzer Plate, and an SMT-120VP Automatic Serum Biochemistry Analyzer (Smart Technology Co., Ltd., Chengdu, China) was used to determine the serum biochemical parameters.

#### 2.4.4. Determination of Antioxidant Function, Immunoglobulin Content, and Cytokine Levels in Serum

Kit-based methods were employed for serum indicator detection. Test kits provided by Shanghai Enzyme-Linked Bio-Tech Co., Ltd. (Shanghai, China) were used for measurement, and the specific experimental steps were strictly followed according to the instructions in the kit manual. The following antioxidant indices were detected: total antioxidant capacity (T-AOC), superoxide dismutase (SOD), catalase (CAT), glutathione peroxidase (GSH-Px), and malondialdehyde (MDA). Immunoglobulins were assessed, including immunoglobulin A (IgA), immunoglobulin G (IgG), and immunoglobulin M (IgM). Cytokine detection included tumor necrosis factor (TNF-α), interleukin-2 (IL-2), interleukin-4 (IL-4), interleukin-6 (IL-6), and interleukin-8 (IL-8). Data for all serum antioxidant, immune, and cytokine parameters were collected only at day 60 of the study. No data for these parameters were collected at day 30.

#### 2.4.5. Rumen Bacteria

Rumen fluid samples, taken from the cryogenic freezer, were placed in 200 mL sterilized triangular flasks, to which 50 mL of PBS buffer (pH 7.2) was added, on 15 September 2023. The samples were then shaken at 200 rpm for 30 min, subjected to ultrasonic treatment at 50 W for 2 min, and subsequently shaken at 150 rpm for another 30 min. The supernatant was collected and transferred to 50 mL sterilized centrifuge tubes that had been previously sterilized under high pressure. The samples were centrifuged at 1500 rpm for 1 min, and the supernatant was transferred to new sterilized 50 mL high-speed centrifuge tubes. The samples were then centrifuged at 12,000 rpm for 10 min to isolate the bacteria. A fast DNA SPIN kit for soil (MP Biomedicals, Solon, OH, USA) was used to extract the DNA from the rumen bacteria. A 1% agarose gel electrophoresis was performed to assess the concentration and purity of the DNA. The V1 and V3 regions of the bacterial 16S rDNA gene were amplified using the primers Ba9F (5′-GAGTTTGATCMTGGCTCAG-3′) and Ba515Rmod1R (5′-CCGCGGCKGCTGGCAC-3′). The PCR amplification system consisted of a 20 µL reaction with the following parameters: 95 °C for 3 min; 94 °C for 20 s, 55 °C for 20 s, and 72 °C for 30 s for 30 cycles; followed by a 10 min extension at 72 °C and then cooled to 10 °C until the reaction stopped. After PCR completion, agarose gel electrophoresis was performed to detect and purify the amplified product. The library was then sent to Meij Biotech for construction and sequenced on the Illumina MiSeq platform using Meij Biotech’s standard protocol for paired-end read lengths of 300 bp.

OTU clustering was performed by clustering sequences into OTUs based on 100% similarity using the RDP classifier for species annotation. The sequences were compared with the Silva 16S rRNA database (v138) using a 70% alignment threshold to obtain microbial taxonomic information at the kingdom, phylum, class, order, family, genus, and species levels. The information was summarized, and subsequent analyses were primarily conducted on the Meijibio Cloud Platform (https://www.majorbio.com, accessed on 31 October 2023). The original data of rumen bacteria in this study can be accessed at https://www.ncbi.nlm.nih.gov/sra/PRJNA1167456, accessed on 31 October 2023, with SRA accession number: PRJNA1167456.

### 2.5. Data Analysis and Statistics

The experimental data were preprocessed using Excel 2018 and analyzed using independent samples *t*-tests with SPSS 20.0 statistical software, except for the bacterial diversity analysis, which was conducted using a one-way ANOVA. Significant differences between treatments were determined using Tukey’s test at *p* < 0.05. The composition and abundance of microorganisms at the phylum and genus levels in rumen fluid were analyzed using the Majorbio Cloud Platform (https://www.majorbio.com, accessed on 31 October 2023).

## 3. Results

### 3.1. Growth Performance

As shown in Table 3, there was no significant difference (*p* > 0.05) in growth performance between the control (CK) group and the *Broussonetia papyrifera* silage-treated (GS) group.

### 3.2. Blood Physiological Indices and Serum Biochemical Indices

As shown in Table 4 and Table 5, on the 30th day of the experiment, the EOS of the GS group was significantly lower (*p* < 0.05) than that of the CK group, and on the 60th day, the NEU, LYM, EOS, and CREA of the GS group were significantly lower (*p* < 0.05) than those of the CK group.

### 3.3. Antioxidant Capacity, Immune Performance, and Cytokines

As shown in Table 6, the antioxidant indicators, immunoglobulin content, and cytokine levels in the GS group were significantly higher (*p* < 0.05) than those in the CK group, while only MDA and IL-4 were significantly lower (*p* < 0.05) than those in the CK group.

### 3.4. Rumen Bacteria

**Figure 1:**
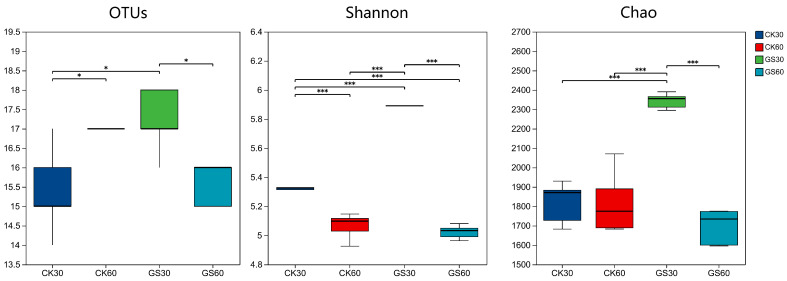
Box plot of α-diversity analysis. CK30 and CK60 represent rumen bacterial samples from the control group at 30 and 60 days, respectively. GS30 and GS60 represent samples from the *Broussonetia papyrifera* silage-treated group at 30 and 60 days, respectively. Significant differences are shown with asterisks: * *p* < 0.05, *** *p* < 0.001.

The diversity of rumen bacteria in the CK group and the GS group was measured using high-throughput analytical approaches. A total of 1,341,945 optimized sequences were obtained. As shown in Figure 1, the GS30 group significantly increased the OTU, Shannon, and Chao indices (*p* < 0.01).

**Figure 2:**
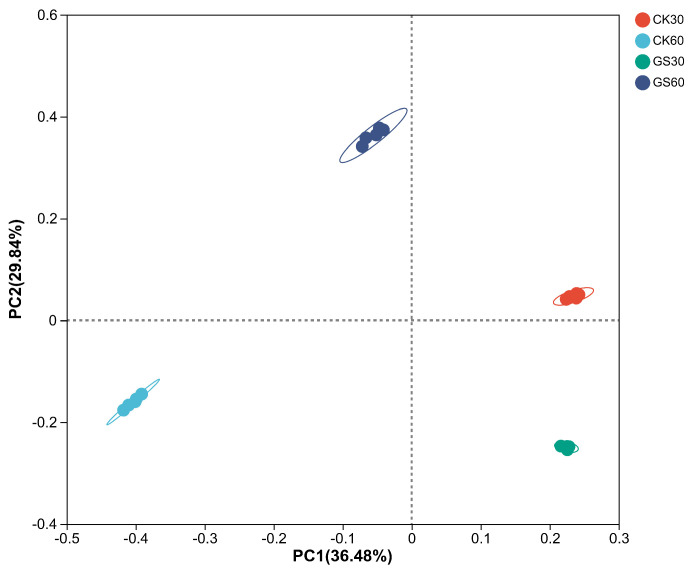
Principal coordinate analysis (PCoA). The axes represent the principal coordinates, with percentages indicating their contribution to sample composition variation. The R-value indicates the between-group difference: a higher R-value means a greater between-group difference.

Using the principal coordinate analysis method, beta-diversity analysis was conducted on rumen microbes, as shown in Figure 2, where there are significant differences in the microbial composition between the treatment groups (*R* = 0.9504, *p* < 0.01).

**Figure 3:**
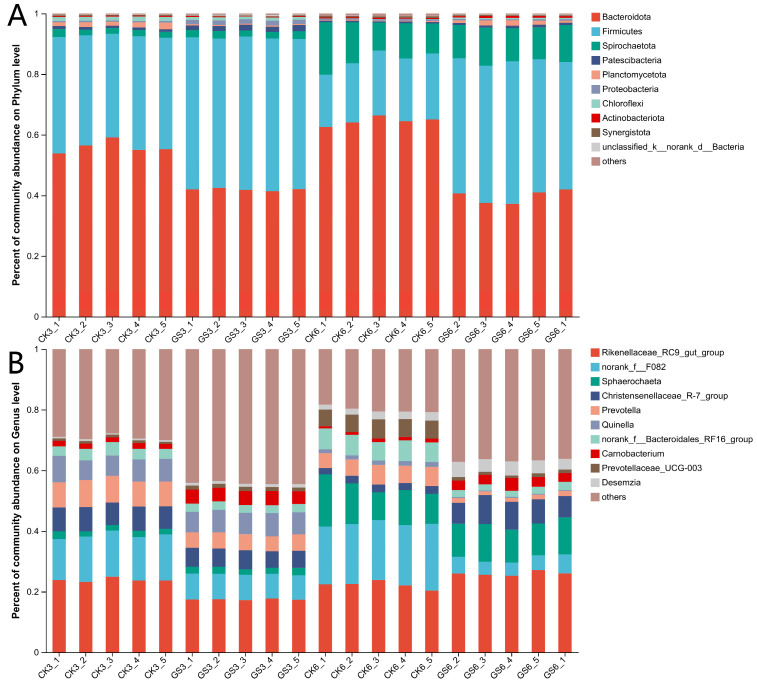
Rumen bacterial communities at the phylum (**A**) and genus (**B**) levels. CK3 and CK6 represent the control group at 30 and 60 days, respectively; GS3 and GS6 represent the *Broussonetia papyrifera*-treated group at 30 and 60 days, respectively.

As shown in Figure 3A, the dominant bacterial phylum in GS3 was *Firmicutes*, with a relative abundance of 49.31% to 50.32%, followed by *Bacteroidota*, which had a relative abundance of 41.41% to 42.42%. In GS6, the dominant phylum remained *Firmicutes*, with a relative abundance of 42.04% to 47.07%, followed by *Bacteroidota* and *Spirochaetota*. In CK3, the dominant phylum was *Bacteroidota*, followed by *Firmicutes*. In CK6, *Bacteroidota* remained dominant, followed by *Firmicutes* and *Spirochaetota*.

As shown in Figure 3B, the dominant bacterial genus in GS3 was *Rikenellaceae_RC9_gut_group*, with a relative abundance of 23.82% to 24.94%, followed by *norank_f__F082* and *Christensenellaceae_R-7_group*. In GS6, *Rikenellaceae_RC9_gut_group* remained dominant, followed by *Sphaerochaeta* and *Christensenellaceae_R-7_group*. In CK3, *Rikenellaceae_RC9_gut_group* had a relative abundance of 17.50% to 17.73%, followed by *norank_f__F082* and *Christensenellaceae_R-7_group*. In CK6, *Rikenellaceae_RC9_gut_group* was dominant, followed by *norank_f__F082* and *Sphaerochaeta*.

**Figure 4:**
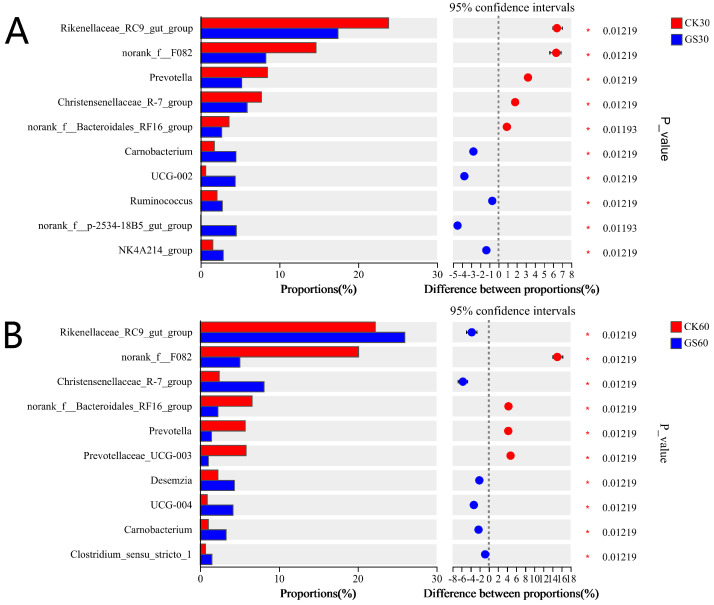
Bar chart of multi-species differences (**A**) at 30 days and (**B**) at 60 days. The X-axis shows different groups, and the Y-axis shows average relative abundance of species.

Compared to the CK30 group (Figure 4A), the GS30 group exhibited significantly higher levels of *Carnobacterium*, *UCG-002*, *Ruminococcus*, *norank_f__p-2534-18B5_gut_group*, and *NK4A2-14_group* (*p* < 0.05). Conversely, levels of *Rikenellaceae_RC9_gut_group*, *norank_f__F082*, *Prevotella*, *Christensenellaceae_R-7_group*, and *norank_f__Bacteroidales_RF16_group* were significantly lower (*p* < 0.05). In comparison with the CK60 group (Figure 4B), the GS60 group showed significantly higher levels of *Rikenellaceae_RC9_gut_group*, *Christensenellaceae_R-7_group*, *Desemzia*, *UCG-004*, *Carnobacterium*, and *Clostridium_sensu_stricto_1* (*p* < 0.05), while *norank_f__F082*, *norank_f__Bacteroidales_RF16_group*, *Prevotella*, and *Prevotellaceae_UCG-003* were significantly lower (*p* < 0.05).

**Figure 5:**
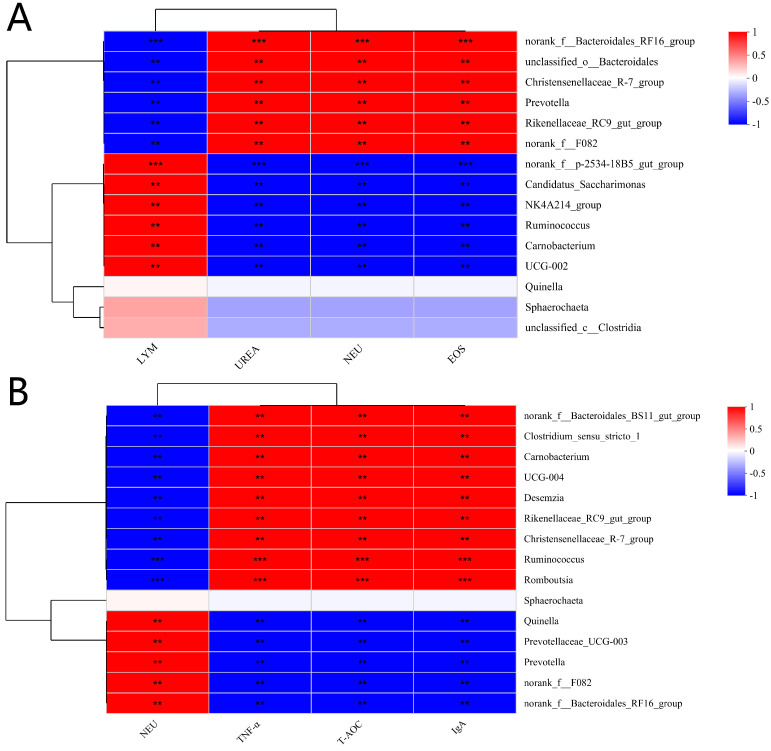
Spearman correlation heatmap: (**A**) 30-day and (**B**) 60-day correlations of rumen bacteria and blood changes. Environmental factors (UREA, LYM, NEU, EOS, TNF-α, T-AOC, IgA) are on the X-axis, and species are on the Y-axis. R-values are color-coded, and statistical significance is indicated by asterisks (** *p* ≤ 0.01, *** *p* ≤ 0.001).

Figure 5A shows that rumen bacteria, including *norank_f__Bacteroidales_RF16_group*, *unclassified_o__Bacteroidales*, *Christensenellaceae_R-7_group*, *Prevotella*, *Rikenellaceae_RC9_gut_group*, and *norank_f__F082*, exhibit strong positive correlations with UREA, NEU, and EOS (*p* < 0.01) and strong negative correlations with LYM (*p* < 0.01). Conversely, *norank_f__p-2534-18B5_gut_group*, *Candidatus_Saccharimonas*, *NK4A2_14_group*, *Ruminococcus*, *Carnobacterium*, and *UCG-002* demonstrate significant negative correlations with UREA, NEU, and EOS (*p* < 0.01) and significant positive correlations with LYM (*p* < 0.01). As shown in Figure 5B, *norank_f__Bacteroidales_BS11_gut_group*, *Clostridium_sensu_stricto_1*, *Carnobacterium*, *UCG-004*, *Desemzia*, *Rikenellaceae_RC9_gut_group*, *Christensenellaceae_R-7_group*, *Ruminococcus*, and *Romboutsia* display significant positive correlations with TNF-α, T-AOC, and IgA (*p* < 0.01) and significant negative correlations with NEU (*p* < 0.01). In contrast, *Quinella*, *Prevotellaceae_UCG-003*, *Prevotella*, *norank_f__F082*, and *norank_f__Bacteroidales_RF16_group* show significant negative correlations with TNF-α, T-AOC, and IgA (*p* < 0.01) and significant positive correlations with NEU (*p* < 0.01).

## 4. Discussion

### 4.1. Growth Performance

*Broussonetia papyrifera* is a fibrous, woody feed characterized by high crude fiber content. The substantial amount of plant fiber not only influences palatability but also impacts the digestion and absorption of nutrients in animals [8]. Our study indicates that feeding a total mixed ration (TMR) containing 20% *B. papyrifera* silage to test animals does not significantly affect their growth performance, possibly due to the similar nutritional levels across the diets. Some studies have reported that incorporating *B. papyrifera* silage into the diets of Lake sheep and their lambs can significantly enhance weight gain [4,7]. Additionally, replacing 15% of the original TMR with *B. papyrifera* silage has been shown to increase the average daily weight gain of beef cattle [8], likely due to the enhanced protein content in the diet. However, other studies indicate that while the inclusion of *B. papyrifera* silage in the diets of Saanen dairy goats does not significantly influence growth performance, it can substantially reduce the cost of weight gain [6]. The varying effects of *B. papyrifera* on animal growth performance may be related to fluctuations in dietary nutrient composition.

Although the addition of 20% *B. papyrifera* silage to the diet did not significantly enhance growth performance in sheep, incorporating hybrid conformation silage as part of a broader feeding strategy may offer economic benefits. The relatively low cost and wide availability of hybrid conformation silage can reduce dependence on more expensive traditional feed sources, improving overall feed cost efficiency and lowering weight gain costs.

### 4.2. Blood Immunoglobulins, Antioxidants, and Cytokines

Blood markers can reveal potential health issues and abnormalities within the body. Feeding sheep and cattle a diet that includes 15% *Broussonetia papyrifera* silage did not significantly affect their blood levels [6,8]. Similar results were observed in this study. However, the levels of neutrophils (NEU), lymphocytes (LYM), eosinophils (EOS), and creatinine (CREA) in the blood of the animals decreased with the inclusion of *B. papyrifera* silage. This effect may be attributed to the rich content of flavonoids and polyphenolic compounds in *B. papyrifera* silage [9,10].

The concentrations of immunoglobulin G (IgG), IgM, and IgA in the body reflect immune function [12]. Numerous studies have demonstrated that feeding *B. papyrifera* silage can enhance the immunoglobulin levels in various animals, including cows, lambs, Dezhou donkeys, rabbits, and pigs [4,13,14,15,16]. In this trial, the serum levels of IgG, IgM, and IgA in the animals significantly increased, indicating that *B. papyrifera* silage can bolster the immune system.

Changes in total antioxidant capacity (T-AOC), superoxide dismutase (SOD), catalase (CAT), glutathione peroxidase (GSH-Px), and malondialdehyde (MDA) are indicative of an animal’s antioxidant capacity. *B. papyrifera* silage is rich in various bioactive compounds, such as unsaturated fatty acids, polyphenolic compounds, terpenoid compounds, and certain alkaloids, which can enhance the activity of SOD, CAT, and GSH-Px while reducing MDA levels [17,18,19,20]. Previous studies have shown that feeding beef cattle, Dezhou donkeys, and piglets *B. papyrifera* silage significantly increases their antioxidant capacity [8,14,16]. The findings of this study align with those of prior research.

Cytokines are essential in regulating immune and inflammatory responses, with pro-inflammatory cytokines like TNF-α, IL-2, IL-6, and IL-8 amplifying immune reactions, while IL-4 typically plays an anti-inflammatory role [21,22]. In this study, the inclusion of *Broussonetia papyrifera* silage in the diet led to increased levels of TNF-α, IL-2, IL-6, and IL-8, indicating the activation of pro-inflammatory pathways. This may be a response to the fibrous nature of the silage or the presence of anti-nutritional factors. The decrease in IL-4 suggests a shift toward a more pro-inflammatory immune profile. These findings point to the possibility that *B. papyrifera* silage influences immune function by modulating cytokine levels, potentially enhancing the animal’s ability to cope with dietary stress or inflammation [23,24]. However, the exact mechanisms underlying these changes are unclear, and further research is needed to explore the impact of *B. papyrifera* silage on immune signaling and overall immune regulation.

In summary, *Broussonetia papyrifera* silage enhances immunity, helps regulate oxidative stress, and reduces inflammation in animals. These benefits contribute to improved overall health and productivity, which, in turn, lower farming costs and reduce livestock mortality.

### 4.3. Rumen Bacteria

The rumen microbial community in ruminants constitutes a complex and dynamic system that not only facilitates the breakdown of feed and nutrient absorption but also plays a crucial role in regulating the immune system and maintaining the stability of the rumen’s internal environment [25]. In this study, feeding *Broussonetia papyrifera* silage resulted in shifts in the rumen microbial community, indicating the potential of the sheep to adapt to the fibrous nature of the diet.

*Firmicutes* are the primary microorganisms responsible for cellulose degradation in the ruminant rumen. The GS group showed a significant increase in the relative abundance of *Firmicutes* while decreasing the relative abundance of *Bacteroidetes*, reflecting the animal’s ability to adjust to and degrade plant fibers in the silage. The observed reduction in *Bacteroidetes* may be attributed to the inhibitory effects of tannins and other anti-nutritional factors present in *Broussonetia papyrifera* [26].

*Rikenellaceae_RC9_gut_group* can neutralize cellular oxidation reactions, thereby protecting cells from oxidative stress and reducing inflammation [27]. Similarly, *Christensenellaceae_R-7_group* is a beneficial bacterium that promotes rumen development and nutrient digestion and absorption [28,29]. At 30 days, the relative abundances of *Rikenellaceae_RC9_gut_group* and *Christensenellaceae_R-7_group* in the GS group were lower than those in the CK group, but by 60 days, these abundances had increased, suggesting that prolonged feeding of *Broussonetia papyrifera* silage may support the adaptation of the sheep’s rumen microbiota to the diet, enhancing beneficial bacterial populations and helping mitigate inflammatory responses.

There is a long-term mutual dependence and regulatory relationship among different microorganisms, and this dynamic interaction is closely related to nutrient absorption and transport in the body. This experiment suggests that feeding *Broussonetia papyrifera* silage may enhance the relative abundances of beneficial genera, including *Carnobacterium*, *Rikenellaceae_RC9_gut_group*, *Christensenellaceae_R-7_group*, and *Desemzia*, which are linked to soluble carbohydrate metabolism, nutrient digestion and absorption, and the host’s antioxidant capacity [28,29,30]. Correlation analysis indicates that these genera are significantly positively correlated with immune and antioxidant indicators in the body. Furthermore, the results show a reduction in the relative abundances of *Quinella*, *Prevotella*, *Sphaerochaeta*, and *Prevotellaceae_UCG-003* in the rumen, genera associated with fiber degradation and methane emissions [29,31,32,33,34].

We observed distinct effects of *Broussonetia papyrifera* silage on the growth performance of ruminants (cattle and sheep). These differences are likely attributed to variations in the digestive systems and dietary needs of cattle and sheep, leading to species-specific responses to the silage [8,12]. Cattle typically have larger rumens and more robust, diverse rumen microbial communities, which enable them to more efficiently degrade plant fibers and absorb greater amounts of nutrients from the silage [35]. In contrast, the rumen microbial shifts observed in sheep in this study suggest adaptive changes in their microbial communities to accommodate the inclusion of *B. papyrifera* silage [36]. Overall, the rumen microbiota of cattle is more diverse and resilient, allowing them to adapt more rapidly to dietary changes, enhancing nutrient absorption and fiber degradation, and potentially mitigating the negative impacts of anti-nutritional factors present in *B. papyrifera* silage [37]. These differences in rumen morphology and microbial communities between cattle and sheep likely explain the observed variation in growth performance when *B. papyrifera* silage is included in the diet.

## 5. Conclusions

*Broussonetia papyrifera* silage can be used to substitute 20% of the daily diet (on a dry matter basis) for Kazakh lamb without negatively impacting their growth performance. Moreover, *B. papyrifera* silage enhances the immune and antioxidant properties of the lamb. Long-term feeding of this silage significantly increases the abundance of beneficial bacteria, such as *Rikenellaceae_RC9_gut_group* and *Christensenellaceae_R-7_group*, in the rumen of Kazakh lamb. Therefore, *Broussonetia papyrifera* silage represents a promising alternative feed resource for ruminants.

## Figures and Tables

**Table 1:** Nutritional levels of *Broussonetia papyrifera* silage and total corn silage (dry matter basis) ^1^.

Item	*Broussonetia papyrifera* Silage	SEM	Total Corn Silage	SEM
pH	4.58	0.004	3.77	0.415
Dry matter	35.11	0.102	34.14	0.289
Crude protein	17.88	0.101	7.34	0.145
Neutral detergent fiber	42.78	0.303	48.21	0.271
Acid detergent fiber	30.16	0.635	22.79	0.364
Water-soluble carbohydrate	8.32	0.106	4.68	0.212
Crude ash	10.42	0.069	4.34	0.087

^1^ n = 4 SEM, standard error of the mean.

**Table 2:** Composition and nutrient levels of experimental diets (DM basis).

Items	Control Group	Experimental Group
Ingredients		
Corn (%)	35.00	35.00
Wheat straw (%)	15.00	15.00
Soybean meal (%)	6.00	6.00
Cottonseed meal (%)	3.00	3.00
Alfalfa hay (%)	15.00	12.00
Total corn silage (%)	22.00	5.00
*Broussonetia papyrifera* silage (%)	-	20.00
NaCl (%)	0.30	0.30
Baking soda (%)	0.70	0.70
Premix ^(1)^	3.00	3.00
Total	100.00	100.00
Nutrient levels		
Metabolic energy (MJ·kg^−1^)	11.15	11.19
Crude protein (%)	14.12	13.92
Neutral detergent fiber (%)	35.55	35.81
Acid detergent fiber (%)	26.30	26.36
Calcium (%)	0.64	0.63
Phosphorus (%)	0.52	0.53

Note (1): per kg of premix contained the following: VA at 3000 IU, VD at 7500 IU, VE at 19 IU, Cu at 18.5 mg, Zn at 118.5 mg, Fe at 81 mg, Mn at 118.5 mg, Se at 0.75 mg, and Co at 1.75 mg. ME was a calculated value, while the others were measured values.

**Table 3:** Effect of *Broussonetia papyrifera* Silage on the Growth Performance of Kazakh lamb.

Item	Groups		SEM	*p*-Value
	CK	GS		
Initial weight (kg)	30.12	30.15	0.038	0.849
Final weight (kg)	37.53	37.62	0.005	0.946
F/G	6.23	6.26	0.122	0.135
ADMI (g/d)	1150.21	1173.20	12.35	0.067
ADG (g/d)	123.50	124.50	0.024	0.125
Carcass weight (kg)	20.64	20.69	0.135	0.237
Carcass yield (%)	55.00	55.00	0.013	0.257

SEM, standard error of the mean. Significant differences were considered as *p* < 0.05. ADG, average daily weight gain. ADMI, average daily feed intake. CK denotes the control group, while GS signifies the treatment group that was administered *Broussonetia papyrifera* silage.

**Table 4:** Effect of *Broussonetia papyrifera* silage on blood parameters of Kazakh lamb after 30 and 60 days.

Item	Time	Groups		SEM	*p*-Value
		CK	GS		
WBC (10^9^/L)	30 d	6.66	6.61	0.159	0.895
	60 d	7.54	6.66	0.380	0.293
NEU (10^9^/L)	30 d	2.94	1.95	0.724	0.556
	60 d	1.95	0.21	0.453	0.029
LYM (10^9^/L)	30 d	3.82	4.73	0.275	0.096
	60 d	4.73	3.68	0.286	0.045
EOS (10^9^/L)	30 d	2.21	0.17	0.562	0.049
	60 d	3.95	0.38	0.871	0.010
RBC (10^12^/L)	30 d	10.41	10.58	0.245	0.765
	60 d	10.65	10.11	0.226	0.276
HGB (g/L)	30 d	120.33	119.00	5.051	0.912
	60 d	108.67	125.00	4.715	0.070
HCT (%)	30 d	32.03	32.57	1.179	0.849
	60 d	31.30	32.70	1.002	0.078
MCV (%)	30 d	30.77	30.70	0.494	0.955
	60 d	30.20	31.33	0.352	0.106
MCH(pg)	30 d	11.53	11.23	0.259	0.620
	60 d	11.07	11.30	0.083	0.184
MCHC (g/L)	30 d	375.33	365.67	8.184	0.613
	60 d	383.33	360.67	7.412	0.134
PLT (10^9^/L)	30 d	550.33	506.00	27.420	0.246
	60 d	610.67	635.33	16.521	0.248
MPV(fL)	30 d	6.27	5.90	0.240	0.508
	60 d	5.67	6.27	0.235	0.236
PDW (fL)	30 d	6.97	7.50	0.687	0.742
	60 d	8.13	6.70	0.601	0.276
PCT (%)	30 d	0.35	0.24	0.044	0.269
	60 d	0.40	0.42	0.044	0.341

SEM, standard error of the mean. Significant differences were considered as *p* < 0.05. White blood cell count (WBC), neutrophil count (NEU), lymphocyte count (LYM), eosinophil count (EOS), red blood cell count (RBC), hemoglobin concentration (HGB), hematocrit (HCT), mean corpuscular volume (MCV), mean corpuscular hemoglobin content (MCH), mean corpuscular hemoglobin concentration (MCHC), platelet count (PLT), mean platelet volume (MPV), platelet distribution width (PDW), and plateletcrit (PCT). CK denotes the control group, while GS signifies the treatment group that was administered *Broussonetia papyrifera* silage.

**Table 5:** Effect of *Broussonetia papyrifera* silage on serum biochemical indicators of Kazakh lamb after 30 d and 60 d.

Item	Time	Groups		SEM	*p*-Value
		CK	GS		
ALB (g/L)	30 d	32.53	32.00	1.233	0.855
	60 d	34.03	34.10	0.762	0.328
TP (g/L)	30 d	75.03	73.40	2.015	0.731
	60 d	70.47	76.20	1.996	0.169
GLOB (g/L)	30 d	42.53	41.37	1.823	0.787
	60 d	38.00	44.73	2.364	0.174
TB (umol/L)	30 d	0.80	3.10	1.275	0.429
	60 d	1.47	0.47	0.309	0.105
AST (U/L)	30 d	90.67	95.33	1.612	0.165
	60 d	90.67	99.33	2.569	0.083
ALT (U/L)	30 d	17.00	17.33	1.327	0.916
	60 d	17.00	19.33	0.833	0.184
AMY (U/L)	30 d	22.33	17.50	5.908	0.729
	60 d	22.33	28.33	4.709	0.584
CK (U/L)	30 d	220.00	121.33	38.583	0.236
	60 d	22.33	28.33	4.709	0.584
Crea (umol/L)	30 d	67.20	59.33	2.726	0.166
	60 d	71.47	58.67	3.024	0.004
UREA (mmol/L)	30 d	7.34	6.60	0.378	0.388
	60 d	7.34	7.39	0.079	0.776
Glu (mmol/L)	30 d	5.08	5.24	0.129	0.586
	60 d	4.35	6.42	0.692	0.146
TG (mmol/L)	30 d	0.40	0.61	0.098	0.344
	60 d	0.30	0.63	0.097	0.077

SEM, standard error of the mean. Significant differences were considered as *p* < 0.05. Albumin (ALB), total protein (TP), globulin (GLOB), total bilirubin (TB), aspartate aminotransferase (AST), alanine aminotransferase (ALT), amylase (AMY), creatine kinase (CK), creatinine (Crea), urea (UREA), glucose (Glu), and triglycerides (TG). CK denotes the control group, while GS signifies the treatment group that was administered *Broussonetia papyrifera* silage.

**Table 6:** Influence of *Broussonetia papyrifera* silage on serum antioxidant activity, immune response, and cytokine profiles of Kazakh lamb after 60 days.

Item	Groups		SEM	*p*-Value
	CK	GS		
T-AOC (pg/mL)	131.81	272.19	16.30	<0.01
SOD (pg/mL)	159.34	288.80	15.16	<0.01
CAT (pg/mL)	623.13	1201.12	66.89	<0.01
GSH-Px (pg/L)	418.14	839.68	49.15	<0.01
MDA (nmol/L)	7.70	2.81	0.57	<0.01
IgA (μg/mL)	122.39	328.33	24.25	<0.01
IgM (μg/mL)	3.55	13.25	1.13	<0.01
IgG (μg/mL)	353.66	1183.82	97.06	<0.01
TNF-α (ng/L)	256.35	953.37	80.56	<0.01
IL-2 (pg/mL)	431.76	1139.45	82.64	<0.01
IL-4 (pg/mL)	158.84	88.35	8.19	<0.01
IL-6 (pg/mL)	69.88	219.56	17.36	<0.01
IL-8 (pg/mL)	14.13	51.60	4.39	<0.01

SEM, standard error of the mean. Significant differences were considered as *p* < 0.05. Total antioxidant capacity (T-AOC), superoxide dismutase (SOD), catalase (CAT), glutathione peroxidase (GSH-Px), malondialdehyde (MDA); immunoglobulin detection: immunoglobulin (IgA), immunoglobulin (IgG), immunoglobulin (IgM); cytokine detection: tumor necrosis factor (TNF-α), interleukin-2 (IL-2), interleukin-4 (IL-4), interleukin-6 (IL-6), interleukin-8 (IL-8). CK denotes the control group, while GS signifies the treatment group that was administered *Broussonetia papyrifera* silage.

## Data Availability

The original contributions presented in the study are included in the article, further inquiries can be directed to the corresponding author.
